# Reduced 8-Gray Compared to Standard 12-Gray Total Body Irradiation for Allogeneic Transplantation in First Remission Acute Lymphoblastic Leukemia: A Study of the Acute Leukemia Working Party of the EBMT

**DOI:** 10.1097/HS9.0000000000000812

**Published:** 2023-01-09

**Authors:** Alexandros Spyridonidis, Myriam Labopin, Bipin Savani, Sebastian Giebel, Gesine Bug, Stefan Schönland, Nicolaus Kröger, Matthias Stelljes, Thomas Schroeder, Andrew McDonald, Igor-Wolfgang Blau, Martin Bornhäuser, Montse Rovira, Wolfgang Bethge, Andreas Neubauer, Arnold Ganser, Jean Henri Bourhis, Matthias Edinger, Bruno Lioure, Gerald Wulf, Kerstin Schäfer-Eckart, Mutlu Arat, Zinaida Peric, Christoph Schmid, Ali Bazarbachi, Fabio Ciceri, Arnon Nagler, Mohamad Mohty

**Affiliations:** 1 Bone Marrow Transplantation Unit and Institute of Cellular Therapy, University of Patras, Greece; 2 Sorbonne University, Saint-Antoine Hospital, AP-HP, INSERM UMRs 938, Paris, France; 3 Vanderbilt University Medical Center, Nashville, TN, USA; 4 Maria Sklodowska-Curie National Research Institute of Oncology, Gliwice, Poland; 5 Department of Medicine II, Hematology, Medical Oncology, Goethe-University, Frankfurt, Germany; 6 Medical Department V, University Hospital Heidelberg, Germany; 7 Department of Stem Cell Transplantation, University Medical Center, Hamburg, Germany; 8 Department of Hematology/Oncology, University of Muenster, Germany; 9 Department of Bone Marrow Transplantation, University Hospital, Essen, Germany; 10 Alberts Cellular Therapy, Netcare Pretoria East Hospital, South Africa; 11 Charité Universitätsmedizin Berlin, Germany; 12 University Hospital, TU Dresden, Germany; 13 BMT Unit, Hematology Department, Institute of Hematology & Oncology, Josep Carreras Institute, Barcelona, Spain; 14 Department of Hematology & Oncology, University Hospital Tuebingen, Medical Center, Tuebingen, Germany; 15 Philipps Universitaet Marburg, University of Hospital Giessen and Marburg, Germany; 16 Hannover Medical School, Germany; 17 Gustave Roussy Cancer Campus BMT, Department of Hematology, Villejuif, France; 18 Department of Hematology and Oncology, University Regensburg, Germany; 19 Hematology, Stem Cell Transplant Unit, ICANS, Strasbourg, France; 20 University Medicine Goettingen, Hematology and Medical Oncology, Goettingen, Germany; 21 Klinikum Nuernberg, 5. Medizinische Klinik, BMT-Unit, Nuernberg, Germany; 22 Demiroglu Bilim Univ. Istanbul Florence Nightingale Hospital, Istanbul, Turkey; 23 University Hospital Centre, Zagreb, Croatia; 24 Universitatsklinikum Augsburg, Section Stem Cell Transplantation, Augsburg University and Medical Faculty, Germany; 25 Bone Marrow Transplantation Program, Department of Internal Medicine, American University of Beirut-Medical Center, Beirut, Lebanon; 26 Ospedale San Raffaele s.r.l., Haematology and BMT, Milano, Italy; 27 Hematology Division, Chaim Sheba Medical Center, Tel-Hashomer, Israel

## Abstract

In this registry-based study, we compared outcomes of allogeneic hematopoietic cell transplantation (allo-HCT) in adult patients with acute lymphoblastic leukemia (ALL) transplanted in first complete remission (CR-1), following conditioning with total body irradiation (TBI) at a standard 12-Gray or at a lower 8-Gray total dose. Patients received fludarabine (flu) as the sole chemotherapy complementing TBI. Eight-Gray TBI/flu was used in 494 patients and 12-Gray TBI/flu in 145 patients. Eighty-eight (23.1%) and 36 (29%) of the patients had Ph-negative B-ALL, 222 (58.3%) and 53 (42.7%) had Ph-positive B-ALL, 71 (18.6%) and 35 (28.2%) T-ALL, respectively (*P* = 0.008). Patients treated with 8-Gray were older than ones received 12-Gray (median 55.7 versus 40.3 years, *P* < 0.0001) and were more frequently administered in vivo T-cell depletion (71% versus 40%, *P* <0.0001). In a multivariate model adjusted for age, type of ALL, and other prognostic factors, leukemia-free survival (primary endpoint) as well as relapse, nonrelapse mortality, overall survival, and GVHD-free, relapse-free survival were not influenced by the TBI dose. These results were confirmed when we focused on patients <55 years of age (median 47 years). Patients with Ph-positive ALL or T-ALL had significantly better survival outcomes than ones with Ph-negative B-ALL, mainly due to significantly fewer relapses. We conclude that 8-Gray TBI is sufficient for adult patients with ALL transplanted in CR-1 with no additional benefit of augmenting the conditioning intensity to 12-Gray.

## INTRODUCTION

Total body irradiation (TBI) is an important part of many preparative regimens used before allogeneic hematopoietic cell transplantation (allo-HCT) and is considered as the standard backbone for conditioning in acute lymphoblastic leukemia (ALL).^1–4^ TBI provides adequate immunosuppression to avoid allograft rejection and efficient antileukemic activity; however, it is associated with considerable acute and long-term toxicity.^5^ While the conditioning effect can be achieved also with low irradiation dosages, the antileukemic activity of TBI is dose-dependent and thus the maximum tolerated dose should be preferentially used.^6,7^ The optimal TBI dose remains in debate, with very few randomized studies on this issue published >30 years ago.^8^ Altogether, 12-Gray (Gy) TBI has been established as the standard dose, as it was shown that further dose escalation does not provide any apparent survival benefit, at least in patients transplanted in first complete remission (CR-1).^6^ A randomized study of acute myeloid leukemia (AML) suggested that reduced intensity conditioning (RIC) with 8-Gy TBI is sufficient and preferable than myeloablative conditioning (MAC) using 12-Gy TBI.^9,10^ Since the issue of using 8-Gy instead of 12-Gy TBI has never been investigated in ALL, we aimed to analyze this using a registry-based large dataset. Besides irradiation dose, the chemotherapy compounds given together with the TBI contribute markedly to overall treatment toxicity and antileukemic activity. Thus, to limit the effect of the chemotherapy counterpart of the conditioning regimen on the analysis of the best TBI dose, we included only patients receiving fludarabine (flu) combined with TBI (TBI/flu) and compared 8- and 12-Gy TBI/flu-treated ALL patients transplanted in CR-1.

## MATERIALS AND METHODS

### Study design and data collection

This is a retrospective, multicenter, registry-based analysis. Data were provided by the Acute Leukemia Working Party (ALWP) of the European Society for Blood and Marrow Transplantation (EBMT) registry in which >600 transplant centers report annually all their consecutive HCTs and according to EBMT-specific quality measures. EBMT centers commit to obtain informed consent according to the local regulations applicable at the time of transplantation and report pseudonymized data to the EBMT. The study was conducted in accordance with the Declaration of Helsinki guidelines. Transplant data from allo-HCTs performed between 2009 and 2020 were screened: from 8978 adult patients with ALL who were allografted in CR-1 (Ph+ B-ALL, n = 2940, 42%; Ph-negative B-ALL, n = 2,132, 31%; T-ALL, n = 1886, 27%; missing, n = 2020), a total of 5792 (64.5%) patients received TBI (at any dose), which was combined either with cyclophosphamide (n = 3842, 60%), with etoposide (n = 736, 12.7%), with other chemotherapy combinations (n = 157, 2.7%) or with flu alone (n = 1057, 18.2%; n = 746, 12.9% with a TBI dose >6-Gy). Patients with the following criteria were included in the analysis: (1) adult (≥18 years) patients diagnosed with ALL; (2) first allogenic peripheral blood or bone marrow HCT in CR-1; (3) human leukocyte antigen (HLA)-matched sibling donor (MSD) or 9–10/10 HLA-matched unrelated donor (UD); (4) conditioning regimen based on TBI at a total dose of either 12- or 8-Gy given in combination with flu (8-Gy TBI/flu versus 12-Gy TBI/flu). Transplantations with ex vivo T-cell depletion were excluded from the analysis (n = 216, 2.3% of 8978 screened patients). Measurable residual disease (MRD) data at HCT were reported by the centers according to their local methodology. The list of institutions reporting data included in this study is given in the Appendix in the Suppl. data.

### Definitions and statistical analysis

The primary objective of the study was the impact of the TBI dose on leukemia-free survival (LFS) defined as time being alive without evidence of relapse (REL). Secondary endpoints included: acute graft-versus-host disease (aGvHD) and chronic GvHD (cGvHD) defined and graded according to standard criteria; nonrelapse mortality (NRM) defined as death without evidence of REL; REL incidence (RI); overall survival (OS) defined as time to death from any cause; and refined GvHD-free, relapse-free survival (GRFS) defined as time being alive with neither grade III-IV aGvHD nor severe cGvHD nor disease REL. Probabilities of OS, LFS, and GRFS were calculated from time of transplant using the Kaplan–Meier estimate.^11^ The follow-up time was calculated using the reverse Kaplan–Meier method. GvHD, RI, and NRM were calculated using cumulative incidence curves in a competing risk setting. Univariate comparisons between groups were performed using the Chi-square and Fisher exact tests for categorical variables and the Mann–Whitney test for continuous variables, the Gray statistic for cumulative incidence functions (GvHD, NRM, REL) and the log-rank test for survival outcomes (OS, LFS, and GRFS). Multivariate analysis was performed using a Cox proportional-hazards model which included variables differing significantly between the groups, factors known to be associated with outcomes, plus a center frailty effect to take account of the heterogeneity across centers. The results were expressed as hazard ratios (HRs) with 95% confidence interval (CIs). All tests were two-sided with the type 1 error rate fixed at 0.05. Statistical analyses were performed with SPSS 27.0 (SPSS Inc., Chicago, IL) and R 4.1.1 (R Development Core Team, Vienna, Austria, URL: https://www.R-project.org/).

## RESULTS

### Characteristics of study population

Baseline patient, transplant and disease characteristics are shown by the study cohort in Table 1. Included in the analysis were 639 patients with ALL (n = 275, 54.5% Ph+ B-ALL, n = 124, 24.6% Ph-negative B-ALL, n = 106, 21.0% T-ALL) who were allografted in CR-1 between 2009 and 2020 and received either an 8-Gy intermediate TBI dose (n = 494) or a 12-Gy standard TBI dose (n = 145) conditioning combined with flu. The distribution of diagnoses differed significantly between the two groups (*P* = 0.008). As expected, patients treated with 8-Gy versus 12-Gy TBI/flu were older (median age 55.7 versus 40.3 years, respectively, *P* < 0.0001), and there was a trend toward a higher proportion of patients with a Karnofsky score <90% (n = 161, 34% versus n = 37, 26%, respectively, *P* = 0.062). The HCT-comorbidity index (HCT-CI) score was available in 330 (66.8%) of patients receiving 8-Gy and in 109 (75.2%) of patients treated with 12-Gy, among which we found a similar distribution of patients with an HCT-CI score of 0 (n = 198, 60% versus n = 67, 61.5%, respectively), 1 or 2 (n = 50, 15.2% versus n = 21, 19.3%, respectively) or ≥3 (n = 82, 24.8% versus n = 21, 19.3%, respectively) (*P* = 0.37). As we included only patients in CR-1, the median time from diagnosis to transplant was short in both groups (<6 months, *P* = 0.27). MRD data at allo-HCT were available for 374 (76%) and 80 (55%) of the patients treated with 8- and 12-Gy, respectively, and among those the percentage with MRD positivity was not significantly different between groups (n = 137, 36.6% versus n = 34, 42.5%, respectively, *P* = 0.33). The most used stem cell source was peripheral blood (>90%) from a UD (in nearly 70% of patients in both groups; 79% 10/10 and 21% 9/10 HLA-matched). Patients in the 8-Gy TBI group received more frequently in vivo T-cell depletion (n = 352, 71.5% versus n = 56, 38.9% in the 12-Gy TBI/flu, *P* < 0.0001). Alemtzumab was used in 25 patients and 383 patients received antithymocyte (ATG) or anti-T lymphocyte (ATLG) globulin (ATG at a dose of 2.5, 5, and ≥7 mg/kg for 1%, 18%, and 7% patients, respectively; ATLG at 20, 30, and >30 mg/kg for 1%, 48%, and 25%, respectively). A combination of 2 immunosuppressive drugs was mostly used as posttransplant GvHD prophylaxis (Suppl. Table S1).

**Table 1:** Patient, Disease, and Transplantation Characteristics

Variable		Total Study Population (n = 639)			Patients < 55 y of Age (n = 360)		
		8-Gy TBI/flu (n = 494)	12-Gy TBI/flu (n = 145)	*P*	8-Gy TBI/flu (n = 229)	12-Gy TBI/flu (n = 131)	*P*
Age (y)	Median (min–max) [IQR]	55.7 (19.8–79.5) [50.2–61.3]	40.3 (18.9–58.7) [27–50.2]	**<0.0001**	49.8 (19.8–55) [43.9–52.7]	35.2 (18.9–54.9) [26.3–46.8]	**<0.0001**
Age classes	Age < 55 years	229 (46.4%)	131 (90.3%)	**<0.0001**	**–**	**–**	**–**
	Age > = 55 years	265 (53.6%)	14 (9.7%)		–	–	–
Year of allo-HCT	Median (min–max)	2017 (2009–2020)	2018 (2009–2020)	**0.001**	2017 (2009–2020)	2018 (2009–2020)	**0.022**
Time diagnosis to HCT (months)	Median (min–max) [IQR]	5.5 (1.9–23) [4.8–6.9]	5.8 (2.8–19.5) [4.5–8.3]	0.27	5.5 (3.2–15.8) [4.8–6.5]	5.8 (2.8–19.5) [4.6–8.5]	0.097
Diagnosis	Ph-negative B-ALL	88 (23.1%)	36 (29%)	**0.008**	43 (22.6%)	35 (31%)	**0.019**
	Ph-positive B-ALL	222 (58.3%)	53 (42.7%)		109 (57.4%)	46 (40.7%)	
	T-ALL	71 (18.6%)	35 (28.2%)		38 (20%)	32 (28.3%)	
	Missing	113	21		39	18	
Sex	Male	275 (55.7%)	86 (59.7%)	0.39	128 (55.9%)	79 (60.8%)	0.37
	Female	219 (44.3%)	58 (40.3%)		101 (44.1%)	51 (39.2%)	
	Missing	0	0		0	1	
Female to male	No	417 (84.4%)	114 (79.7%)	0.18	192 (83.8%)	103 (79.2%)	0.27
	Yes	77 (15.6%)	29 (20.3%)		37 (16.2%)	27 (20.8%)	
	Missing	0	2		0	1	
Donor type	MSD	140 (28.3%)	47 (32.4%)	0.34	80 (34.9%)	40 (30.5%)	0.39
	UD	354 (71.7%)	98 (67.6%)		149 (65.1%)	91 (69.5%)	
Stem cell source	Bone marrow	36 (7.3%)	8 (5.5%)	0.46	19 (8.3%)	8 (6.1%)	0.45
	Peripheral blood	458 (92.7%)	137 (94.5%)		210 (91.7%)	123 (93.9%)	
MRD at HCT	Negative	237 (63.4%)	46 (57.5%)	0.33	111 (63.8%)	40 (56.3%)	0.28
	Positive	137 (36.6%)	34 (42.5%)		63 (36.2%)	31 (43.7%)	
	Missing	120	65		55	60	
Karnofsky score	<90	161 (34%)	37 (25.7%)	0.062	87 (39.2%)	32 (24.6%)	**0.005**
	> = 90	313 (66%)	107 (74.3%)		135 (60.8%)	98 (75.4%)	
	Missing	20	1		7	1	
Patient CMV	Negative	210 (42.7%)	55 (38.5%)	0.37	105 (45.9%)	52 (40.3%)	0.31
	Positive	282 (57.3%)	88 (61.5%)		124 (54.1%)	77 (59.7%)	
	Missing	2	2		0	2	
Donor CMV	Negative	263 (53.9%)	57 (40.1%)	**0.004**	120 (52.9%)	52 (40.6%)	**0.027**
	Positive	225 (46.1%)	85 (59.9%)		107 (47.1%)	76 (59.4%)	
	Missing	6	3		2	3	
In vivo TCD	No	140 (28.5%)	88 (61.1%)	**<0.0001**	71 (31.1%)	81 (62.3%)	**<0.0001**
	Yes	352 (71.5%)	56 (38.9%)		157 (68.9%)	49 (37.7%)	
	Missing	2	1		1	1	
Engraftment	Graft Failure	8 (1.6%)	1 (0.7%)	0.69	4 (1.8%)	1 (0.8%)	0.66
	Engrafted	477 (98.4%)	140 (99.3%)		219 (98.2%)	127 (99.2%)	
	Missing	9	4		6	3	

Significant P values in tables are given in bold.

ALL = acute lymphoblastic leukemia; allo-HCT = allogeneic hematopoietic cell transplantation; CI = confidence interval; CMV = cytomegalovirus; flu = fludarabine; GvHD = Graft versus Host Disease; GRFS = GvHD-free, relapse-free survival; Gy = Gray; HR = hazard ratio; IQR = interquartile range; LFS = leukemia-free survival; MRD = measurable residual disease; MSD = matched sibling donor; n = number of patients; NRM = nonrelapse mortality; OS = overall survival; Ph = Philadelphia chromosome; REL = relapse; RI = relapse incidence; TBI = total body irradiation; TCD = T-cell depletion; UD = unrelated donor.

### TBI schedule

The TBI schedule was not reported for 155 (31.3%) and 27 (18.6%) patients receiving 8- and 12-Gy TBI, respectively. Assuming that missing values could be inferred in centers having always reported the same number of fractions, we were able to estimate the fractionation mode in most patients (n = 572, 89%), thus having missing data in only 44 (8.9%) of patients treated with 8-Gy and in 23 (15.8%) of patients treated with 12-Gy TBI/flu. A delivered dose of ≤2 Gy per fraction was the most frequently used modality both for patients receiving 8-Gy TBI (n = 288, 85%; estimated n = 393, 87.3%) or 12-Gy TBI (n = 89, 75.4%; estimated n = 93, 76.2%). Fractions of 2.7–4 Gy were delivered in 51 (15%; estimated n = 57, 12.6%) and 23 (19.5%; estimated n = 23, 18.8%) patients treated with 8- and 12-Gy, respectively. Six (5.1%) patients received 2 boosts of 6-Gy (results not shown).

### Univariate comparison between 8- and 12-Gy TBI groups

**Figure 1.**
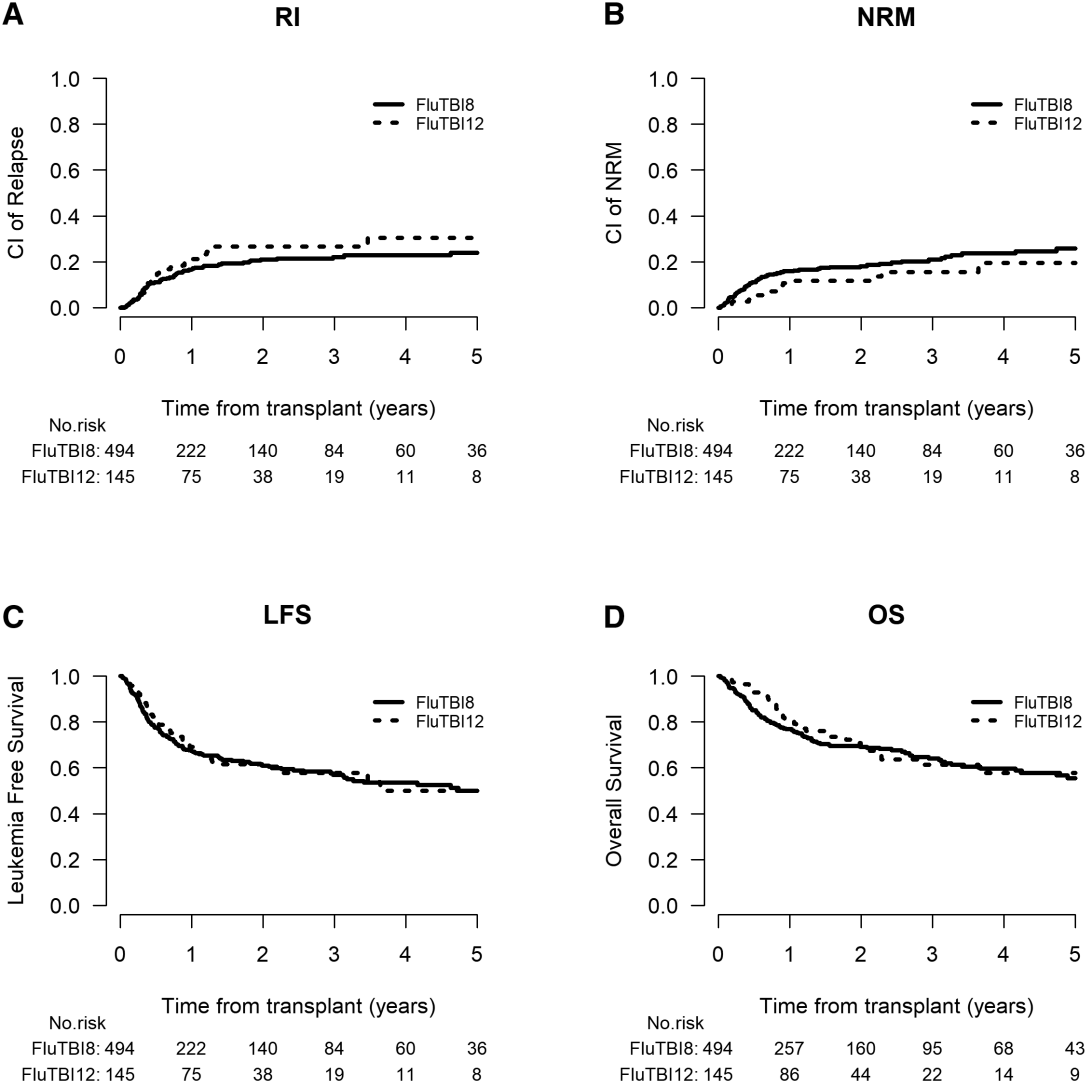
**Outcomes of all patients according to the TBI dose.** LFS = leukemia-free survival; NRM = nonrelapse mortality; OS = overall survival; RI = relapse incidence; TBI = total body irradiation.

In the univariate comparison (Table 2), there was no significant difference in LFS or other outcomes (OS, GRFS) in relation to the TBI dose used, with the corresponding survival curves being superimposable (Figure 1). In particular, the LFS rates were 60.9% (55.6–65.7) and 61.7% (51.9–70) in the 8- and 12-Gy groups, respectively (*P* = 0.41). No significant differences in the cumulative incidence of aGvHD (II-IV or III-IV), cGvHD (overall or extensive), REL or NRM was found between patients treated with 8- and 12-Gy TBI (Table 2). Significant covariates of outcomes in the univariate analysis were found to be patient age (associated with NMR, LFS, OS, GRFS), type of ALL (with REL, LFS, OS, GRFS), type of donor (REL, grade II-IV aGvHD, overall and extensive cGvHD and GRFS), and use of in vivo TCD (with overall and extensive cGvHD and GRFS) (results not shown). The main causes of death were disease recurrence (n = 55, 40.7% versus n = 76, 43.9%), infection (n = 36, 26.7% versus n = 41, 23.7%), and GvHD (n = 17, 12.6% versus n = 22, 12.7%), respectively. Other causes of death did not differ between groups (Suppl. Table S2).

**Table 2:** Cumulative Incidence (95% CI) of GvHD and 2-Year Survival Outcomes for the Whole Cohort and for Patients < 55 Years of Age

Outcome	Total Study Population (n = 639)			Patients < 55 y of Age (n = 360)		
	8-Gy TBI/flu (n = 494)	12-Gy TBI/flu (n = 145)	*P*	8-Gy TBI/flu (n = 229)	12-Gy TBI/flu (n = 131)	*P*
Acute GvHD, II-IV (180 d)	26.7% (22.8–30.7)	23.6% (16.8–31.1)	0.5	23% (17.7–28.8)	24.9% (17.7–32.8)	0.7
Acute GvHD, III-IV (180 d)	8.8% (6.5–11.5)	8.1% (4.3–13.5)	0.85	6.8% (4–10.7)	8.8% (4.7–14.6)	0.46
Chronic GvHD (2 y)	39.6% (34.3–44.8)	36.8% (27.5–46.1)	0.48	39% (31.1–46.9)	39% (28.8–49)	1
Extensive chronic GvHD (2 y)	16.1% (12.3–20.2)	15.1% (9–22.7)	0.6	14.7% (9.6–20.7)	17.3% (10.3–25.9)	0.85
REL	21% (16.9–25.3)	26.7% (18.8–35.1)	0.19	22.4% (16.3–29.2)	23.7% (15.7–32.6)	0.96
NRM	18.2% (14.5–22.2)	11.7% (6.7–18.3)	0.056	11.7% (7.3–17.2)	12.2% (6.8–19.3)	0.87
LFS	60.9% (55.6–65.7)	61.7% (51.9–70)	0.65	65.8% (57.8–72.7)	64.1% (53.7–72.7)	0.91
OS	69.1% (64–73.7)	69.3% (59–77.5)	0.41	72.8% (65–79.2)	70.9% (59.6–79.5)	0.83
GRFS	47.7% (42.4–52.8)	50% (40.3–59)	0.44	53.1% (45.1–60.5)	50.9% (40.5–60.5)	0.97

CI = confidence interval; flu = fludarabine; GvHD = Graft versus Host Disease; GRFS = GvHD-free, relapse-free survival; Gy = Gray; HR = hazard ratio; IQR = interquartile range; LFS = leukemia-free survival; n = number of patients; NRM = nonrelapse mortality; OS = overall survival; Ph = Philadelphia chromosome; REL = relapse; TBI = total body irradiation.

### Factors found to influence outcomes in the multivariate analysis

In the multivariate analysis adjusted for age and variables differing significantly between the two TBI groups (complete case analysis including diagnosis, n = 484), TBI dose was not found to significantly influence LFS (HR 1.18; 95% CI, 0.73-1.93; *P* = 0.5). Administration 8- or 12-Gy was not found to be an independent prognostic factor for aGvHD (all grades), cGvHD (all grades), REL or NRM, nor to affect probabilities of OS and GRFS (Table 3 and Suppl. Table S3). As expected, an incremental age was associated with increased NRM (HR 1.66; 95% CI, 1.25-2.22; *P* = 0.0005) and lower LFS (HR 1.24; 95% CI, 1.05-1.47; *P* = 0.011), OS (HR 1.32; 95% CI, 1.09-1.59; *P* = 0.004), and GRFS (HR 1.19; 95% CI, 1.04-1.37; *P* = 0.013). The use of in vivo TCD reduced cGvHD (HR 0.4; 95% CI, 0.27-0.59; *P* < 0.0001) and NRM (HR 0.5; 95% CI, 0.29-0.86; *P* = 0.012) translating into an improved GRFS (HR 0.66; 95% CI, 0.48-0.9; *P* = 0.008). Type of ALL significantly influenced outcomes. When compared to patients with Ph-negative B-ALL (referent group), patients with Ph-positive B-ALL and T-ALL had a lower risk of REL (HR 0.43; 95% CI, 0.26-0.7; *P* < 0.0008 and HR 0.43; 95% CI, 0.24-0.8; *P* < 0.007, respectively) translating into better LFS (HR 0.5; 95% CI, 0.35-0.73; *P* < 0.0003 and HR 0.51; 95% CI, 0.32-0.81; *P* < 0.005, respectively) and OS (HR 0.49; 95% CI, 0.32-0.74; *P* < 0.0007 and HR 0.5; 95% CI, 0.3-0.85; *P* < 0.01, respectively). In addition, diagnosis of Ph-positive ALL was associated with reduced rates of cGvHD (HR 0.52; 95% CI, 0.34-0.8; *P* < 0.003) and better GRFS (HR 0.57; 95% CI, 0.41-0.79; *P* < 0.0007) when compared to Ph- ALL.

**Table 3:** Factors Found to Influence Outcomes in the Multivariate Analysis (Complete Case Analysis)

Factor	Total Study Population (n = 484 With Complete Information)		Patients < 55 y of Age (n = 292 With Complete Information)	
	HR (95% CI)	*P*	HR (95% CI)	*P*
Relapse				
12- vs 8-Gy TBI/flu	1.09 (0.56–2.14)	0.79	0.62 (0.28–1.38)	0.24
Ph+ B-ALL (ref. Ph− ALL)	**0.43 (0.26–0.7**)	**0.0008**	**0.46 (0.25–0.86**)	**0.015**
T-ALL (ref. Ph− B-ALL)	**0.43 (0.24–0.8**)	**0.007**	**0.42 (0.19–0.9**)	**0.027**
NRM				
12- vs 8-Gy TBI/flu	1.17 (0.54–2.54)	0.68	0.95 (0.35–2.58)	0.93
Age (per 10 y)	**1.66 (1.25–2.22**)	**0.0005**	**1.79 (1.1–2.9**)	**0.018**
In vivo TCD	**0.5 (0.29–0.86**)	**0.012**	**0.25 (0.09–0.64**)	**0.004**
LFS				
12- vs 8-Gy TBI/flu	1.18 (0.73–1.93)	0.5	0.83 (0.46–1.53)	0.56
Age (per 10 y)	**1.24 (1.05–1.47**)	**0.011**	1.05 (0.82–1.34)	0.71
Ph+ B-ALL (ref. Ph− ALL)	**0.5 (0.35–0.73**)	**0.0003**	**0.57 (0.34–0.95**)	**0.031**
T-ALL (ref. Ph− B-ALL)	**0.51 (0.32–0.81**)	**0.005**	**0.5 (0.27–0.96**)	**0.036**
OS				
12- vs 8-Gy TBI/flu	1.34 (0.81–2.2)	0.25	0.9 (0.48–1.69)	0.74
Age (per 10 y)	**1.32 (1.09–1.59**)	**0.004**	1.1 (0.84–1.46)	0.48
Ph+ B-ALL (ref. Ph− ALL)	**0.49 (0.32–0.74**)	**0.0007**	0.56 (0.31–1.01)	0.054
T-ALL (ref. Ph− B-ALL)	**0.5 (0.3–0.85**)	**0.01**	0.61 (0.3–1.25)	0.18
GRFS				
12- vs 8-Gy TBI/flu	1.08 (0.73–1.6)	0.69	0.82 (0.48–1.39)	0.46
Age (per 10 y)	**1.19 (1.04–1.37**)	**0.013**	1.05 (0.85–1.29)	0.68
Ph+ B-ALL (ref. Ph− ALL)	**0.57 (0.41–0.79**)	**0.0007**	**0.62 (0.39–0.98**)	**0.042**
In vivo TCD	**0.66 (0.48–0.9**)	**0.008**	**0.53 (0.33–0.85**)	**0.009**
Acute GvHD II-IV				
12- vs 8-Gy TBI/flu	0.81 (0.44–1.47)	0.48	0.87 (0.44–1.72)	0.69
Chronic GvHD				
12- vs 8-Gy TBI/flu	0.81 (0.49–1.35)	0.42	0.82 (0.45–1.52)	0.54
Ph+ B-ALL (ref. Ph− ALL)	**0.52 (0.34–0.8**)	**0.003**	**0.53 (0.29–0.94**)	**0.031**
In vivo TCD	**0.4 (0.27–0.59**)	**<0.0001**	**0.3 (0.17–0.56**)	**0.0001**
Year HCT	**0.92 (0.86–0.99**)	**0.032**	0.92 (0.82–1.03)	0.14

Significant P values in tables are given in bold.

ALL = acute lymphoblastic leukemia; allo-HCT = allogeneic hematopoietic cell transplantation; CI = confidence interval; CMV = cytomegalovirus; flu = fludarabine; GvHD = Graft versus Host Disease; GRFS = GvHD-free, relapse-free survival; Gy = Gray; HR = hazard ratio; IQR = interquartile range; LFS = leukemia-free survival; MRD = measurable residual disease; MSD = matched sibling donor; n = number of patients; NRM = nonrelapse mortality; OS = overall survival; Ph = Philadelphia chromosome; REL = relapse; RI = relapse incidence; TBI = total body irradiation; TCD = T-cell depletion; UD = unrelated donor.

### Patients <55 years of age

**Figure 2.**
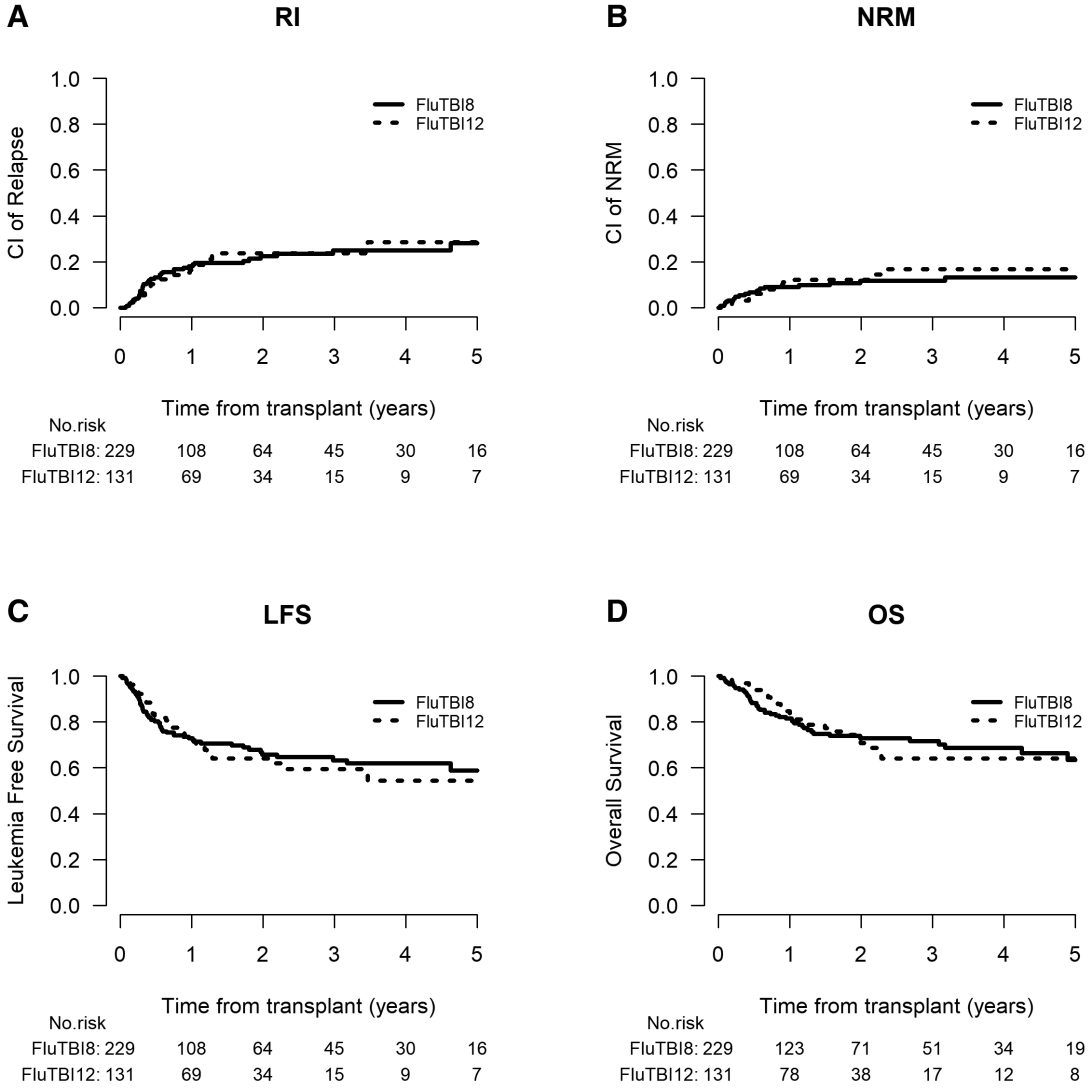
**Outcomes of patients < 55 y of age according to the TBI dose.** LFS = leukemia-free survival; NRM = nonrelapse mortality; OS = overall survival; RI = relapse incidence; TBI = total body irradiation.

There was an unequal distribution of patients aged below or equal to or above 55 years between the TBI groups (*P* < 0.0001) with only 14 patients (9.6%) of the patients treated with 12-Gy TBI/flu being ≥55 years of age, whereas in the 8-Gy TBI/flu group, the proportion of patients aged < or ≥55 years was similar (n = 229, 46.4% versus n = 265, 53.6%, respectively) (Table 1). To minimize the obvious bias of being ≥55 years of age and not being treated with the higher 12-Gy TBI dose, we focused on patients <55 years of age (n = 360, median 47 years). Two hundred twenty-nine patients were conditioned with 8-Gy TBI/flu and 131 patients received 12-Gy TBI/flu (Figure 2). Both univariate and multivariate analysis (complete case analysis including diagnosis, n = 292) showed no influence of the TBI dose on LFS [8-Gy, 65.8% (57.8-72.7) versus 12-Gy, 64.1% (53.7-72.7), *P* = 0.91; HR 0.83; 95% CI, 0.46-1.53; *P* = 0.56] nor on other outcomes (REL, NRM, GVHD, OS, GRFS) (data shown in Tables 2 and 3; Suppl. Table S4). The negative effect of increased age on LFS, OS, and GRFS observed in the entire population was attenuated when focusing only on patients <55 years of age. As in the total study population, both diagnosis and in vivo TCD remained significant covariates in the multivariate analysis of the younger patients. When compared to Ph-negative B-ALL, patients with Ph-positive B-ALL had a significantly lower risk of REL and cGvHD (HR 0.46; 95% CI, 0.25-0.86; *P* = 0.015 and HR 0.53; 95% CI, 0.29-0.94; *P* = 0.031, respectively), better GRFS and LFS (HR 0.62; 95% CI, 0.39-0.98; *P* = 0.042 and HR 0.57; 95% CI, 0.34-0.95; *P* = 0.031, respectively) and a trend toward a better OS (HR 0.56; 95% CI, 0.31-1.01; *P* = 0.054). T-ALL was associated with a lower REL risk (HR 0.42; 95% CI, 0.19-0.9; *P* = 0.027) and better LFS (HR 0.5; 95% CI, 0.27-0.96; *P* = 0.036). Administration of in vivo TCD was associated with a lower risk of NRM (HR 0.25; 95% CI, 0.09-0.64; *P* = 0.004), lower cGvHD rates (HR 0.3; 95% CI, 0.17-0.56; *P* = 0.0001) and improved GRFS (HR 0.53; 95% CI, 0.33-0.85; *P* = 0.009).

## DISCUSSION

In this registry-based study, we compared for the first-time outcomes of allo-HCT for adults with ALL in CR-1 using TBI at a standard 12-Gy or at a lower 8-Gy total dose. The multivariate analysis demonstrated that LFS (primary endpoint) and other outcomes (GvHD, REL, NRM, OS, GRFS) were not influenced by the irradiation dose. These results were similar when we focused on patients <55 years of age.

Although not examined in randomized trials in adults, TBI-based regimens are considered to be preferable for conditioning in ALL.^2,12^ TBI was used in approximately 80% of 13,460 allogeneic transplants for adult patients with ALL reported in the EBMT registry between 2001 and 2015^13^ and was still frequently used in more recent years (2009–2015) also for patients transplanted in CR-1 (in 64.5% of 8,978 transplants). TBI schedules range from a single dose between 2 and 10 Gy to a fractionated dose of 8–15.75 Gy once- or twice-daily over 3–4 days.^14,15^ According to a survey performed among EBMT centers, the 12-Gy total dose is the most commonly used.^14^ Historically, increasing the TBI dose from 12 to 15.75 Gy significantly reduced the probability of posttransplant REL, but did not improve survival because of increased mortality from other causes.^16^ Lowering the total TBI dose from 12- to 8-Gy to reduce short- and long-term irradiation toxicity has been attempted in pediatric patients with hematologic malignancies with good outcomes.^17,18^ The comparison of 8-Gy TBI to the standard 12-Gy TBI has never been the subject of a prospective or retrospective study in adult ALL patients.

A German study group compared prospectively 2 different irradiation conditioning regimens (TBI 12-Gy/cyclophosphamide versus TBI 8-Gy/flu) for AML patients transplanted in CR-1 and found no differences in the REL incidence and a tendency toward reduced NMR with the 8-Gy TBI regimen.^9,10^ However, besides the lower TBI dose, the substitution of cyclophosphamide with the less toxic flu may have led to the reduced NRM. In ALL, TBI-based conditioning regimens most frequently include cyclophosphamide, etoposide and/or flu. From these, flu has apparently contributed less to overall treatment toxicity and antileukemic activity.^19^ A recent EBMT analysis in adult patients with ALL allografted in CR has found that the use of cyclophosphamide in 12-Gy TBI regimens is associated with a stronger antileukemic effect when compared to 12-Gy TBI/flu (Giebel et al EBMT 48th annual Meeting, abstr. OS16-04). Thus, to limit the effect of the chemotherapy counterpart in our analysis asking whether 8-Gy TBI is sufficient for ALL patients transplanted in CR-1, we decided to include only patients who received flu as the sole chemotherapy counterpart of TBI. Besides TBI dose, the method of TBI delivery may affect both safety and efficacy and varies among centers.^14^ Fractionated TBI schedules have been adopted as optimal schedules in HCT and are used most frequently, at least over the last two decades.^6,14^ In fact, we were able to capture the TBI schedule in nearly 90% of the patients confirming that the 2-Gy fractionated mode was used in most of them (n = 486, 85%). Eighty patients (14%) received the total TBI dose in fractions of 4 Gy, which in retrospective analyses had similar outcomes with the 2-Gy fractionation.^20^ Taken together, our analysis which included patients treated with fractionated TBI/flu could focus on the effect of the irradiation dose on outcomes.

One would expect the lower TBI dose to result in less GvHD and reduced NRM, something which was not found here. One major limitation in this type of analysis is that the patient populations are fundamentally different, as older, and more comorbid patients were precluded from having a higher TBI dose. Still, age-adjusted multivariate analysis of the whole study population as well as the analysis of only those <55 years of age did not suggest that reduction of the delivered TBI dose would result in lower GvHD and NRM rates. Advances in supportive care and radiation delivery in recent years may have resulted in a reduced NRM with the higher TBI dose similar to the lower 8-Gy dose.^21^ Indeed, the 11.7% NRM rate found in the group of patients treated with 12-Gy TBI/flu (median year of transplant 2018) is considered to be relative low.^13^

An important question is whether lowering the TBI dose would have any effect on residual disease clearance and REL rates. The inverse correlation between conditioning intensity and REL rates has been clearly demonstrated in patients with myeloid malignancies^22^ and has been suggested in multiple retrospective analyses in patients with ALL comparing RIC versus MAC.^23,24^ In the age-adjusted Cox analysis for the whole population as well as for the <55 years of age patients, REL rates were not influenced by the TBI dose. Thus, in patients with ALL transplanted with low-level residual disease (CR-1) the use of higher doses of TBI may be obsolete. As the use of cyclophosphamide in TBI regimens has been found to be associated with significantly reduced relapse rates when compared to TBI/flu (Giebel et al, under revision), we re-run the multivariable Cox model including posttransplant cyclophosphamide as covariate and TBI dose did not influence relapse, both in the entire and in the <55 years of age population. In our cohort, patients diagnosed with Ph-negative B-ALL had higher REL rates and impaired LFS and OS as compared to patients with Ph-positive B-ALL and T-ALL. We assume an impact of pre- and posttransplant tyrosine kinase inhibitor treatment in Ph-positive patients on the reduced REL rates.^25^ Posttransplant tyrosine kinase inhibitor treatment may have also contributed to the reduced cGvHD and better GRFS which was found to be associated with a Ph-positive diagnosis.^26^

Considering the absence of any impact of TBI dose on GvHD, NRM, and REL, it is not surprising that there was no significant difference in LFS and other survival outcomes (OS, GRFS) between patients treated with 12- and 8-Gy TBI. 12-Gy TBI was traditionally defined as MAC and fractionated 8-Gy TBI as RIC.^27,28^ Recently, the ALWP of the EBMT proposed the transplant conditioning intensity (TCI) score for finer stratification of the conditioning regimens in predicting NRM and REL.^29^ Regimen intensity is captured by assigning weight scores for each of the conditioning components and using their sum to generate the TCI score. According to the proposed algorithm, both 8-Gy TBI/flu and 12-Gy TBI/flu regimens fall within the intermediate “reduced-toxicity” conditioning category (TCI 2.5–3.5), in which the traditionally defined RIC and MAC regimens do not differ regarding NRM and REL risk. Consistent with that, we found no impact of the type of conditioning regimen on both NRM and REL.

Our study has all the inherent limitations of a retrospective registry-based analysis. Although we focused on a relatively uniform patient population (ALL, CR-1, TBI/flu only) and tried to overcome further heterogeneity through multivariate modeling, there were still incomplete data (eg, MRD data and methodology) and unmeasured factors (eg, technical aspects of the TBI procedure such as organ shielding, dose rate and dosimetry, patient immobilization, source of radiation, craniospinal boosting) that could not be captured and adjusted for. More importantly, TBI dose and age were linked, and this could only be partially overcome by separately analyzing the younger patients (aged <55 years). Moreover, there was an unequal distribution of diagnoses and use of in vivo TCD across dose levels and both factors were significant covariates in the multivariate analysis. As Ph-positive disease and in vivo TCD had a higher prevalence in the 8-Gy TBI group, we cannot exclude that post-TKI treatment mitigated a negative effect of in vivo T-cell depletion on REL rates. Nevertheless, the multivariate analysis confirmed the consensus-based recommendation of the use of in vivo T-cell depletion to prevent cGVHD and improve GRFS also in first remission ALL.^30^

In conclusion, this is the first large patient series analysis demonstrating a “non-inferiority” of 8-Gy as compared to the standard 12-Gy TBI in adult patients with ALL transplanted in CR-1, a finding that should be validated in prospective trials. Assuming that the lower TBI dose is associated with reduced early toxicity and morbidity, our findings support the use of 8-Gy TBI for ALL in CR-1, especially in elderly or frail patients.^31^ Whether this is also true for patients with more advanced disease (≥CR-2) or young-adults cannot be answered, as our study included only CR-1 patients, few of whom were below 25 years of age.

## ACKNOWLEDGMENTS

We thank the ALWP-EBMT staff for data management. The study was accomplished thanks to the contributing centers of the EBMT registry which provided patient data; a complete list appears in the Suppl. Appendix. Principal investigators at the centers recruiting the largest numbers of patients were included in the authorship.

## DISCLOSURES

The authors have no conflicts of interest to disclose.

## SOURCES OF FUNDING

Article Publication Charges are funded by the Institute of Cell Therapy, University Research Center, University of Patras.

## Supplementary Material
