# Supplementary material for: Reduced 8-Gray Compared to Standard 12-Gray Total Body Irradiation for Allogeneic Transplantation in First Remission Acute Lymphoblastic Leukemia: A Study of the Acute Leukemia Working Party of the EBMT
Source: Hemasphere. 2023 Jan 9;7(1):e812. doi: 10.1097/HS9.0000000000000812 (PMC9831184; doi:10.1097/HS9.0000000000000812)
Supplement: Supplementary file 1 [file hs9-7-e812-s001.docx]

**Supplementary Table 1. GvHD prophylaxis**

| **Causes of Death** | **8-Gy TBI/flu (n=494)** | **12-GY TBI/flu (n=145)** |
| --- | --- | --- |
| Cyclosporin + MTX | 313 (63.6%) | 30 (20.8%) |
| Cyclosporin + MMF | 105 (21.3%) | 43 (29.9%) |
| Cyclosporin alone | 19 (3.9%) | 12 (8.3%) |
| Cyclosporin + Tacro | 9 (1.8%) | 23 (16%) |
| Tacro + MMF | 16 (3.3%) | 17 (11.8%) |
| Tacro + MTX | 10 (2%) | 0 (0%) |
| Tacro + Sirolimus | 1 (0.2%) | 1 (0.7%) |
| Sirolimus + MMF | 3 (0.6%) | 0 (0%) |
| Cyclosporin + Tacro + MMF | 0 (0%) | 2 (1.4%) |
| Cyclosporin + MTX + MMF | 4 (0.8%) | 0 (0%) |
| Other | 12 (2.4%) | 16 (11.1%) |
| Missing | 2 | 1 |
| no ATG no PTCy | 123 (25%) | 22 (15.3%) |
| ATG | 350 (71.1%) | 33 (22.9%) |
| campath | 2 (0.4%) | 23 (16.0%) |
| PTCy | 17 (3.5%) | 66 (45.8%) |

**Supplementary Table 2. Causes of death**

| **Causes of Death** | **8-Gy TBI/flu (n=143)** | **12-GY TBI/flu (n=38)** |
| --- | --- | --- |
| Original disease | 55 (40.7%) | 76 (43.9%) |
| Infection | 36 (26.7%) | 41 (23.7%) |
| GvHD | 17 (12.6%) | 22 (12.7%) |
| Multiorgan Failure | 2 (1.5%) | 3 (1.7%) |
| Non HCT related | 11 (8.1%) | 14 (8.1%) |
| CNS toxicity | 5 (3.7%) | 7 (4%) |
| Other second malignancy | 4 (3%) | 4 (2.3%) |
| Other HCT related | 3 (2.2%) | 3 (1.7%) |
| Hemorrhage | 1 (0.7%) | 1 (0.6%) |
| Veno-occlusive Disease (VOD) | 1 (0.7%) | 2 (1.2%) |
| Missing | 8 | 9 |

**Supplementary Table 3. Multivariate analysis of the entire population**

| **Whole study cohort** | **RELAPSE** | | **NRM** | | **LFS** | | **OS** | | **GRFS** | | **Acute GvHD II-IV** | | **Chronic GvHD** | |
| --- | --- | --- | --- | --- | --- | --- | --- | --- | --- | --- | --- | --- | --- | --- |
| **VARIABLE** | **HR**  **(95% CI)** | **p** | **HR**  **(95% CI)** | **p** | **HR**  **(95% CI)** | **p** | **HR**  **(95% CI)** | **p** | **HR**  **(95% CI)** | **p** | **HR**  **(95% CI)** | **p** | **HR**  **(95% CI)** | **p** |
| **12-Gy vs 8-Gy TBI/flu** | 1.09  (0.56-2.14) | 0.79 | 1.17 (0.54-2.54) | 0.68 | 1.18 (0.73-1.93) | 0.5 | 1.34 (0.81-2.2) | 0.25 | 1.08 (0.73-1.6) | 0.69 | 0.81 (0.44-1.47) | 0.48 | 0.81 (0.49-1.35) | 0.42 |
| Age (per 10y) | 1.04  (0.84-1.28) | 0.74 | **1.66**  **(1.25-2.22)** | **0.0005** | **1.24**  **(1.05-1.47)** | **0.011** | **1.32**  **(1.09-1.59)** | **0.004** | **1.19**  **(1.04-1.37)** | **0.013** | 1.04 (0.87-1.23) | 0.69 | 1.08 (0.91-1.29) | 0.36 |
| Ph-neg. B-ALL (reference) | 1 |  | 1 |  | 1 |  | 1 |  | 1 |  | 1 |  | 1 |  |
| Ph-pos. B-ALL | **0.43**  **(0.26-0.7)** | **0.0008** | 0.65 (0.36-1.17) | 0.15 | **0.5**  **(0.35-0.73)** | **0.0003** | **0.49**  **(0.32-0.74)** | **0.0007** | **0.57**  **(0.41-0.79)** | **0.0007** | 0.98 (0.63-1.52) | 0.93 | **0.52**  **(0.34-0.8)** | **0.003** |
| T-ALL | **0.43**  **(0.24-0.8)** | **0.007** | 0.65 (0.31-1.35) | 0.25 | **0.5**  **(0.32-0.81)** | **0.005** | **0.5**  **(0.3-0.85)** | **0.01** | 0.69 (0.46-1.02) | 0.06 | 1.44 (0.86-2.41) | 0.16 | 0.7 (0.42-1.15) | 0.16 |
| Year HCT | 1 (0.9-1.1) | 0.94 | 0.96 (0.87-1.07) | 0.5 | 0.99 (0.92-1.06) | 0.69 | 0.95 (0.88-1.03) | 0.21 | 0.97 (0.92-1.03) | 0.37 | 1.04 (0.96-1.13) | 0.37 | **0.92**  **(0.86-0.99)** | **0.032** |
| Time diagnosis to HCT | 0.95 (0.87-1.04) | 0.25 | 1.02 (0.94-1.11) | 0.58 | 0.98 (0.93-1.04) | 0.58 | 0.97 (0.91-1.04) | 0.45 | 0.99 (0.94-1.04) | 0.59 | 1.02 (0.96-1.08) | 0.58 | 0.97 (0.91-1.04) | 0.41 |
| Female D to Male R | 0.86 (0.5-1.49) | 0.6 | 1.4 (0.78-2.53) | 0.26 | 1.07 (0.72-1.6) | 0.73 | 1.13 (0.73-1.75) | 0.59 | 1.12 (0.79-1.58) | 0.53 | 1.05 (0.65-1.71) | 0.84 | 0.82 (0.52-1.28) | 0.37 |
| Karnofsky >=90 | 1.27 (0.78-2.07) | 0.34 | 1.03 (0.62-1.72) | 0.9 | 1.19 (0.84-1.69) | 0.34 | 1.07 (0.74-1.56) | 0.72 | 1.33 (0.98-1.79) | 0.064 | 1.2 (0.82-1.76) | 0.36 | 1.18 (0.82-1.7) | 0.38 |
| UD vs MSD | 0.72 (0.44-1.18) | 0.2 | 1.25 (0.69-2.25) | 0.46 | 0.84 (0.58-1.22) | 0.37 | 0.84 (0.56-1.27) | 0.41 | 0.91 (0.66-1.24) | 0.55 | 1.63 (1-2.65) | 0.05 | 0.91 (0.61-1.35) | 0.64 |
| in vivo TCD | 0.85 (0.49-1.49) | 0.58 | **0.5**  **(0.29-0.86)** | **0.012** | 0.72 (0.49-1.07) | 0.1 | 0.78 (0.52-1.16) | 0.21 | **0.66**  **(0.48-0.9)** | **0.008** | 0.9 (0.56-1.44) | 0.66 | **0.4**  **(0.27-0.59)** | **< 0.0001** |
| centre (frailty term) |  | 0.062 |  | 0.93 |  | 0.22 |  | 0.92 |  | 0.26 |  | 0.07 |  | 0.23 |

**Supplementary Table 4. Multivariate analysis of patients < 55 years of age**

| **Cox <55y age** | **RELAPSE** | | **NRM** | | **LFS** | | **OS** | | **GRFS** | | **Acute GVHD II-IV** | | **chronic GVHD** | |
| --- | --- | --- | --- | --- | --- | --- | --- | --- | --- | --- | --- | --- | --- | --- |
| **VARIABLE** | **HR (95% CI)** | **p** | **HR (95% CI)** | **p** | **HR (95% CI)** | **p** | **HR (95% CI)** | **VARIABLE** | **HR (95% CI)** | **p** | **HR (95% CI)** | **p** | **HR (95% CI)** | **p** |
| **12-Gy vs 8-Gy TBI/flu** | 0.62 (0.28-1.38) | 0.24 | 0.95 (0.35-2.58) | 0.93 | 0.83 (0.46-1.53) | 0.56 | 0.9 (0.48-1.69) | 0.74 | 0.82 (0.48-1.39) | 0.46 | 0.87 (0.44-1.72) | 0.69 | 0.82 (0.45-1.52) | 0.54 |
| Age (per 10y) | 0.8 (0.6-1.08) | 0.15 | **1.79**  **(1.1-2.9)** | **0.018** | 1.05 (0.82-1.34) | 0.71 | 1.1 (0.84-1.46) | 0.48 | 1.05 (0.85-1.29) | 0.68 | 0.96 (0.73-1.25) | 0.74 | 1.14 (0.88-1.48) | 0.31 |
| Ph-neg. B-ALL (reference) | 1 |  | 1 |  | 1 |  | 1 |  | 1 |  | 1 |  | 1 |  |
| Ph-pos. B-ALL | **0.46**  **(0.25-0.86)** | **0.015** | 0.88 (0.35-2.23) | 0.79 | **0.57**  **(0.34-0.95)** | **0.031** | 0.56 (0.31-1.01) | 0.054 | **0.62**  **(0.39-0.98)** | **0.042** | 1.31 (0.69-2.46) | 0.41 | **0.53**  **(0.29-0.94)** | **0.031** |
| T-ALL | **0.42**  **(0.19-0.9)** | **0.027** | 0.84 (0.26-2.73) | 0.77 | **0.5**  **(0.27-0.96)** | **0.036** | 0.61 (0.3-1.25) | 0.18 | 0.6 (0.35-1.05) | 0.073 | 1.51 (0.73-3.12) | 0.27 | 0.8 (0.41-1.55) | 0.5 |
| Year HCT | 1.1 (0.94-1.28) | 0.23 | 1.01 (0.82-1.23) | 0.95 | 1.06 (0.94-1.2) | 0.31 | 0.99 (0.87-1.13) | 0.93 | 0.99 (0.9-1.09) | 0.89 | 1.1 (0.96-1.25) | 0.16 | 0.92 (0.82-1.03) | 0.14 |
| Time diagnosis to HCT | 1.03 (0.93-1.14) | 0.53 | 1.07 (0.93-1.24) | 0.36 | 1.05 (0.96-1.13) | 0.28 | 0.96 (0.86-1.07) | 0.46 | 1.02 (0.95-1.1) | 0.6 | 0.97 (0.87-1.08) | 0.55 | 0.99 (0.89-1.1) | 0.89 |
| Female D to Male R | 0.98 (0.48-2) | 0.96 | 2.13 (0.94-4.83) | 0.069 | 1.33 (0.78-2.28) | 0.3 | 1.3 (0.71-2.39) | 0.39 | 1.32 (0.82-2.14) | 0.25 | 1.36 (0.72-2.58) | 0.34 | 0.63 (0.33-1.22) | 0.17 |
| Karnofsky >=90 | 1.6 (0.84-3.05) | 0.16 | 0.55 (0.26-1.15) | 0.11 | 1.03 (0.64-1.66) | 0.9 | 0.87 (0.52-1.43) | 0.58 | 1.45 (0.94-2.23) | 0.093 | 0.9 (0.54-1.5) | 0.69 | 1.09 (0.66-1.78) | 0.74 |
| UD vs MSD | 0.71 (0.38-1.33) | 0.29 | 2.53 (0.99-6.43) | 0.052 | 0.99 (0.59-1.64) | 0.96 | 1.15 (0.64-2.04) | 0.65 | 1.15 (0.74-1.8) | 0.53 | 1.59 (0.85-2.98) | 0.15 | 0.98 (0.57-1.68) | 0.94 |
| in vivo TCD | 1.02 (0.51-2.04) | 0.95 | **0.25**  **(0.09-0.64)** | **0.004** | 0.69 (0.4-1.19) | 0.18 | 0.6 (0.34-1.07) | 0.086 | **0.53**  **(0.33-0.85)** | **0.009** | 0.73 (0.39-1.37) | 0.33 | **0.3**  **(0.17-0.56)** | **0.0001** |
| Centre (frailty term) |  | 0.15 |  | 0.95 |  | 0.19 |  | 0.33 |  | 0.11 |  | 0.12 |  | 0.15 |

**Abbreviations for all Tables**: allo-HCT, allogeneic hematopoietic cell transplantation; ALL, acute lymphoblastic leukemia; CI, confidence interval; CMV, cytomegalovirus; flu, fludarabine; GvHD, Graft versus Host Disease; GRFS, GvHD-free, relapse-free survival; Gy, Gray; HR, hazard ratio; IQR, interquartile range; LFS, leukemia-free survival; MRD, minimal residual disease; MSD: matched sibling donor; n: number of patients; NRM, non-relapse mortality; OS, overall survival; Ph: Philadelphia chromosome; PTCY: post-transplant cyclophosphamide; REL, relapse; RI, relapse incidence; TBI, total body irradiation; TCD, T-cell depletion; UD, unrelated donor; y, year. Significant p values in tables are given in bold.

**Supplementary Appendix. Contributing EBMT centers**

| 524 Heidelberg [Medizinische Kl] | 348 Aachen [RWTH] | 253 Nantes [Hotel Dieu] | 119 Ascoli_Piceno [Osp Mazzoni] |
| --- | --- | --- | --- |
| 614 Hamburg [Univ H] | 513 Munich [Kl Grosshadern] | 518 Berlin [HELIOS Kl] | 125 Dortmund [St. Johannes H] |
| 680 Muenster [University] | 534 Cologne [Univ, Medicine] | 533 Jena [Friedrich-Schiller] | 146 Stuttgart [Diakonissen Kh] |
| 259 Essen [Univ H] | 267 Pessac [H Haut-Leveque] | 661 Rennes [H Sud/Pontchaillou] | 153 Hamburg [AK St Georg] |
| 456 Pretoria [Albert Albert] | 277 Lille [H Claude Huriez] | 141 Brescia [Civili, Adulti] | 204 Ulm [Innere Med III] |
| 807 Berlin [Charité Univ] | 338 Halle [Univ Martin-Luther] | 233 Besancon [H Jean Minjoz] | 232 Rome [Emat, La Sapienza] |
| 297 Frankfurt am Main [Goethe-Univ] | 428 Gliwice [Sklodowska] | 234 Brussels [St. Luc] | 251 Caen [Hopital, Hematol] |
| 808 Dresden [Universitaets Kl] | 558 Munich [Rechts der Isar] | 261 Geneva [261] | 252 Creteil [H Mondor Hematol] |
| 214 Barcelona [H Clinic] | 729 Hradec_Králové [Charles U H, Hem] | 390 Duesseldorf [H Heine U] | 265 Milano [Osp Maggiore] |
| 223 Tuebingen [Univ] | 134 Bonn [Uni] | 665 Clamart [H Percy] | 273 Clermont-Ferrand [Jean Perrin] |
| 645 Marburg [Philipps Univ] | 152 Augsburg [Zentral Kl] | 926 Montpellier [University] | 286 Pavia [S Matteo] |
| 295 Hannover [Medical Univ] | 246 Rotterdam [Erasmus MC] | 190 Frankfurt (Oder) [Clin Internal Med] | 332 Taranto [Osp Nord] |
| 666 Villejuif [Gustave Roussy] | 270 Grenoble [H A Michallon] | 294 Milano [Osp Niguarda] | 502 Venezia [SS Giovani e Paolo] |
| 787 Regensburg [University] | 307 Rome [Univ S Cuore] | 308 Graz [Medical Univ] | 512 Cape_Town [UCT Medical Sc] |
| 672 Strasbourg [H Hautepierre] | 311 Wiesbaden [Kl Diagnostik] | 359 Magdeburg [vGuericke U] | 585 Rostock [Kl Inn. Medicine] |
| 552 Goettingen [Univ Kl] | 656 Prague [Ist Hematology] | 367 Luebeck [Schleswig-Holstein] | 622 Athens [Evangelismos H] |
| 625 Nuernberg [Klinikum] | 658 Bergamo [Ospedale, ematol] | 447 Schwerin [Helios Kl] | 659 Brest [C.H.R.U Brest] |
| 994 Istanbul [Nightingale] | 671 Lyon [H E Herriot] | 523 Nice [H de l`ARCHET I] | 712 Wuerzburg [Medizinische Kl II] |
| 145 Stuttgart [Robert_Bosch_Kh] | 786 Mainz [Johannes-Gutenberg] | 594 Linz [Elisabethinen H] | 726 Liege [University] |
| 207 Paris [St Louis] | 227 Vienna [Medizinische Univ] | 597 Brno [Univ H] | 794 Perugia [Monteluce] |
| 556 Budapest [National Med Ctr] | 256 Kiel [UKSH] | 650 Angers [CHRU] | 970 Flensburg [St Franziskus] |
| 676 Vandoeuvre_Les_Nancy [Hosp] | 624 Toulouse [H Purpan] | 784 Essen [Evangelisches Kh] |  |
| 955 Amiens [H Sud] | 809 Erlangen [University] | 785 Homburg [Univ Saarland] |  |
| 142 Mannheim [Univ] | 160 Paris [H Necker] | 810 Freiburg [University] |  |
| 262 Paris [Pitie-Salpetriere] | 202 Basel [202] | 977 Limoges [CHRU] |  |
